# Transmission enhancement based on strong interference in metal-semiconductor layered film for energy harvesting

**DOI:** 10.1038/srep29195

**Published:** 2016-07-12

**Authors:** Qiang Li, Kaikai Du, Kening Mao, Xu Fang, Ding Zhao, Hui Ye, Min Qiu

**Affiliations:** 1 State Key Laboratory of Modern Optical Instrumentation, College of Optical Science and Engineering, Zhejiang University, Hangzhou 310027, China

## Abstract

A fundamental strategy to enhance optical transmission through a continuous metallic film based on strong interference dominated by interface phase shift is developed. In a metallic film coated with a thin semiconductor film, both transmission and absorption are simultaneously enhanced as a result of dramatically reduced reflection. For a 50-nm-thick Ag film, experimental transmission enhancement factors of 4.5 and 9.5 are realized by exploiting Ag/Si non-symmetric and Si/Ag/Si symmetric geometries, respectively. These planar layered films for transmission enhancement feature ultrathin thickness, broadband and wide-angle operation, and reduced resistance. Considering one of their potential applications as transparent metal electrodes in solar cells, a calculated 182% enhancement in the total transmission efficiency relative to a single metallic film is expected. This strategy relies on no patterned nanostructures and thereby may power up a wide spectrum of energy-harvesting applications such as thin-film photovoltaics and surface photocatalysis.

Interference has been investigated since the 19th century already and have been well described in detail^1^,^2^. Since Capasso group’s report in ref. 3, the strong interference effects in highly absorbing media has attracted significant attention^4^,^5^,^6^,^7^,^8^,^9^,^10^,^11^,^12^,^13^,^14^,^15^,^16^,^17^,^18^,^19^,^20^,^21^,^22^,^23^,^24^,^25^. The strong interference in metal-semiconductor double-layered films has been demonstrated to realize high absorption at optical frequencies^5^,^6^,^7^,^8^. In sheer contrast with conventional interference relying on transmission phase shift, the non-trivial phase shift (not 0° or 180°) at the metal-semiconductor interface dominates and correspondingly a strong resonance can be created in the semiconductor film markedly thinner than light wavelength. Besides tunable absorption^9^,^10^,^11^,^12^,^13^,^14^, other functions in this metal/semiconductor double-layered films with strong interference have also been demonstrated, including thermal emission^15^,^16^, color display^17^,^18^,^19^, enhanced THz radiation^20^,^21^. By further exploiting this strong interference in metal/semiconductor/metal triple-layered films, a Fabry-Perot narrowband color filter has also been demonstrated^22^,^23^,^24^,^25^.

In this paper, we demonstrate that optical transmission can be significantly enhanced relying on this strong interference dominated by interface phase shift in planar metal/semiconductor double-layered films. While the absorption is significantly boosted, the transmission is also remarkably augmented at slightly red-shifted wavelength. Metallic films with simultaneous superior electrical conductivity and high optical transmissivity are highly desirable in many energy-harvesting optoelectronic devices such as liquid-crystal displays, solar cells, light-emitting diodes^26^,^27^. Since no patterned nanostructure is required in this scheme, this method presents the virtue of large-area and lithography-free fabrication as well as design flexibility; besides, the strong interference dominated by interface phase shift reduces the film thickness required for creating resonances and results into a broadband operation and high angular tolerance. This scheme opens an extraordinary transmission window for a seamless metallic layer without any assistance of patterned nanostructures and thus may find potential applications in energy-harvesting applications such as thin-film photovoltaics and surface photocatalysis.

## Results and Discussions

### Ag/Si double layered film

**Figure 1:**
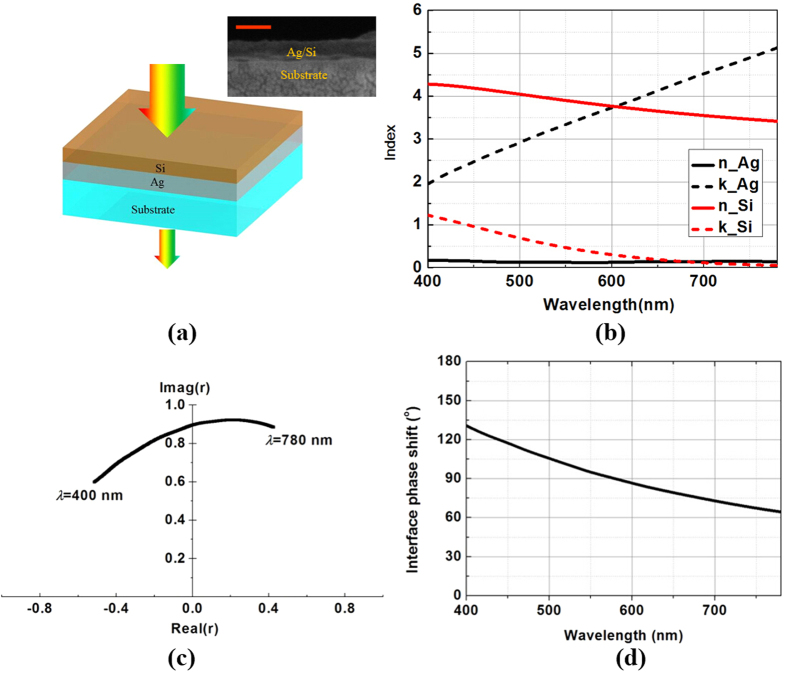
(**a**) A representation (both schematic diagram and SEM image of cross-section) of the double-layered film with enhanced transmission, comprising a high refractive index dielectric layer on an optically thin metallic film on a glass substrate. The scale bar is 100 nm. (**b**) Real and imaginary parts of the complex refractive indices of Ag and Si. (**c**) Real and imaginary parts and (**d**) phase shift (in degree) of the complex reflection coefficient at Ag/Si interface at normal incidence.

To demonstrate the concept of enhanced optical transmission based on strong interference effect in a thin planar metal/semiconductor double-layered film, a semiconductor layer (such as Si, Ge and GaAs) is deposited on a metallic (such as Au, Ag, Al and Cu) layer to create strong interference in between, as illustrated in Fig. 1. The whole structure is placed on a transparent glass substrate (*n* = 1.45). Here amorphous silicon (a-Si) and Ag are used as semiconductor and metal materials, respectively. Figure 1a also presents the SEM image of the Ag/Si double layered film. The boundary between the substrate layer and Ag layer is clear. Figure 1b presents measured real and imaginary parts of the complex refractive indices of sputtered Ag and a-Si using a spectroscopic ellipsometer. The a-Si is highly absorbing at visible frequencies owing to direct electronic transitions at energies above the absorption edge. The Ag thickness is controlled to be below 60 nm. The a-Si thickness *h* ranges from 10 to 25 nm; therefore, *h* ≪ *λ*/4*n* (*λ* is the light wavelength and *n* is the real part of the complex refractive index) is satisfied in the visible range. This ultra-thin Si layer markedly alters the reflection as well as the transmission of the Ag film. The transmission peak wavelength can be altered by varying the Si layer thickness. At optical frequencies from 400 to 780 nm, the metal has a finite conductivity and corresponding a finite complex refractive index (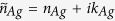) while the semiconductor exhibits a high refractive index together with a high loss (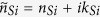). Therefore, a non-trivial phase shift can be formed at the interface. Figure 1c,d present real and imaginary parts and phase shift (in degree) of the complex reflection coefficient 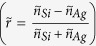 at Ag/Si interface at normal incidence. It can be seen that significant imaginary parts exist in the complex reflection coefficient. Subsequently, the Ag/Si interface phase shift is around 90° from 400 to 780 nm, in sheer contrast with conventional interface phase shift (0° or 180°) between two lossless dielectrics. This non-trivial Ag/Si interface phase shift combines the Air/Si and Ag/SiO_2_ interface phase shifts and propagation phase shifts to create absorption and transmission resonances for certain semiconductor film thicknesses below *λ*/4*n*.

**Figure 2:**
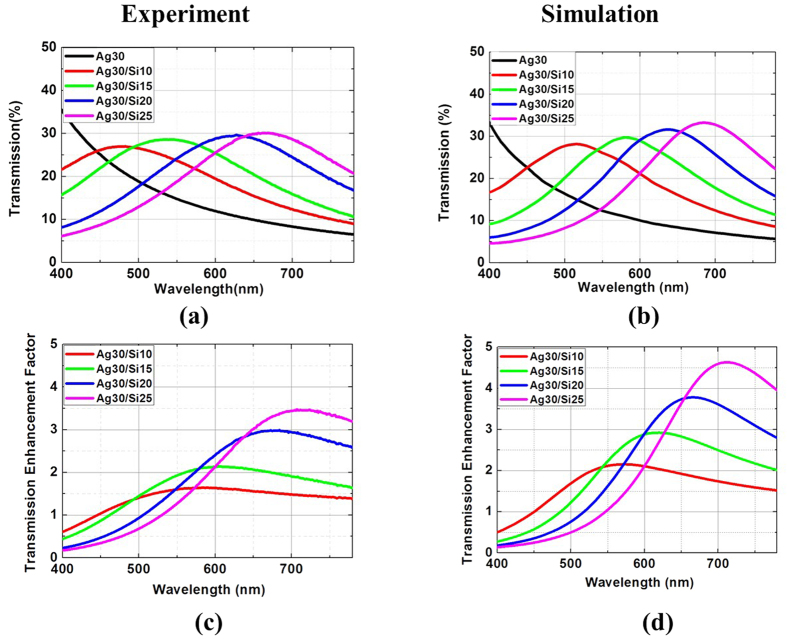
Measured (**a**) transmission and (**c**) transmission enhancement factor at normal incident angle, respectively. (**b**,**d**) Are corresponding simulation results.

To explore the enhanced transmission based on strong interference dominated by interface phase shift in planar metal/semiconductor double-layered films, Si layers with thicknesses of 10–25 nm (in a 5 nm increment) on 30-nm-thick Ag layers are fabricated and experimental transmission at normal incidence in the visible range (400 nm to 780 nm) is provided in Fig. 2a. The transmission photographs of the fabricated Ag/Si double-layered films on 1 cm × 1 cm glass substrate are displayed in Fig. S1 in the Supplementary Materials. The experimental transmission for Ag/Si double-layered films with other Ag thicknesses (20, 40 and 50 nm) is presented in Fig. S2 in the Supplementary Information. The transmission enhancement factor is defined as the transmission of the Ag/Si double-layered film relative to that of a single Ag film. For a single 30-nm-thick Ag film, the measured transmission drops at increasing wavelength and is below 10% beyond 650 nm wavelength. Once a thin Si layer is covered, a notable resonant enhanced transmission relative to that of the referenced Ag film can be clearly distinguished. The enhanced transmission features a broadband operation. For a 15-nm-thick Si coating, the transmission is enhanced beyond 460 nm wavelength and the measured transmission maximum is 28% at 550 nm, which almost doubles in comparison with that of a single Ag film. By controlling the Si thickness, the strong interference and consequently transmission characteristics of the double-layered film can be manipulated. The transmission peak wavelength red-shifts as the Si thickness is increased. The peak wavelength shifts to around 670 nm when the Si thickness is increased to 25 nm. The transmission enhancement is more significant for a thick Ag film. Specifically, for a 50-nm-thick Ag film at 670 nm, the transmission is increased from 1.3% to 6.3% after coating a 25-nm-thick Si film, exhibiting an over fourth enhancement in the transmission (Figs S2d and S3d). Using the measured optical constants of a-Si and Ag, the calculated transmission and transmission enhancement factor corresponding to the measurements are presented in Fig. 2c,d, respectively. Excellent agreement is obtained between the experimental data and the calculations. The slightly low transmission in experiments can be attributed to the light scattering loss induced by the rough film surfaces.

**Figure 3:**
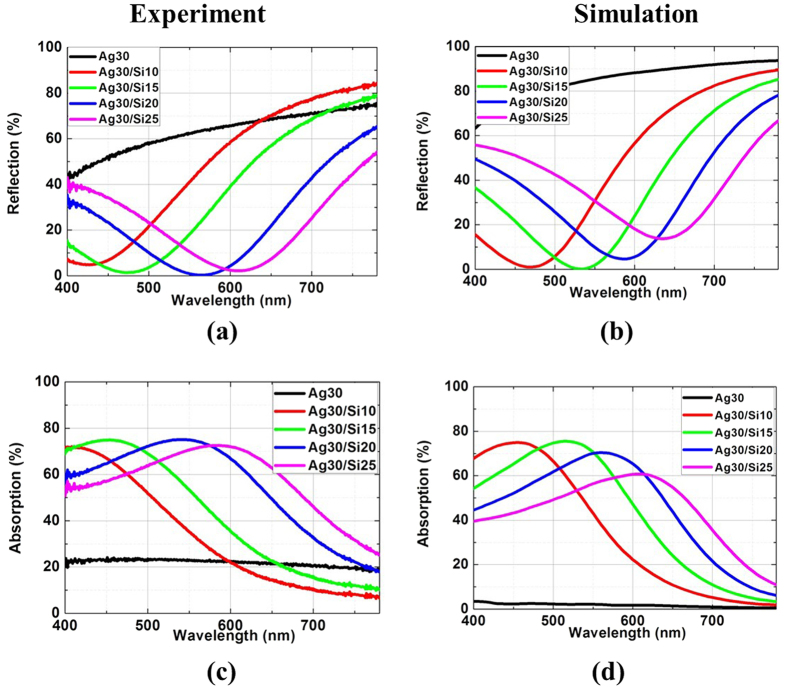
Measured (**a**) reflection and (**c**) absorption at normal incident angle, respectively. (**b**,**d**) are corresponding simulation results.

The transmission is directly related to the reflection and the absorption. To further explore their correlation, measured and simulated reflection (*R*) and absorption (*A* =1−*R*−*T*) of Ag/Si double-layered films at normal incidence for 30-nm-thick Ag are presented in Fig. 3. The results for Ag/Si double-layered films with other Ag thicknesses (20, 40 and 50 nm) are presented in Figs S4 and S5 in the Supplementary Materials. For single 30-nm-thick Ag film, the measured reflection increases with wavelength and reaches 75% at 780 nm wavelength while the absorption is around 20% from 400 to 780 nm wavelength. While the transmission is significantly enhanced resulting from the strong interference after the Si layer is imposed, the absorption is also remarkably augmented. For a 15-nm-thick Si coating, the measured reflection is reduced to nearly null at 470 nm, which is slightly shorter than peak transmission wavelength (around 550 nm). Meanwhile, the peak absorption is dramatically increased to above 70% at 470 nm, well surpassing that of a single Ag film (around 20%). Therefore, the significantly reduced reflection contributes to enhanced transmission as well as enhanced absorption.

**Figure 4:**
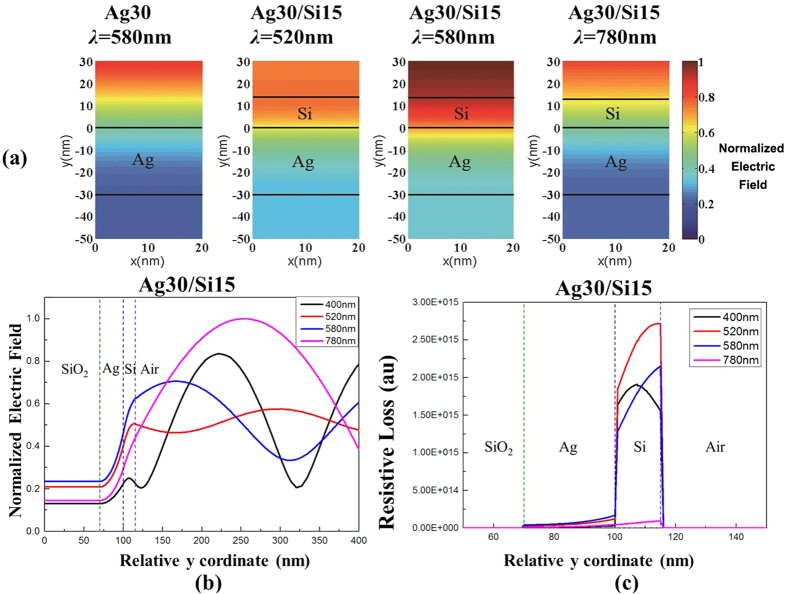
(**a**) Contour plot of simulated electric field profile across and above the devices for Ag30 at *λ* = 580 nm and for Ag30/Si15 at *λ* = 580 nm and 450 nm. (**b**,**c**) Are electric field profile and resistive loss profile across Ag30/Si15 devices for four typical wavelength (*λ* = 400/520/540/580/780 nm).

To unveil the physics behind the enhanced transmission, Fig. 4a,b provide the contour plots of simulated electric field profile and corresponding electric field value across all layers at several operating wavelength (including simulated peak transmission wavelength 580 nm, peak absorption wavelength 520 nm, non-resonant wavelength 780 nm) for a Ag/Si (30/15 nm) double-layered film, respectively. For a single 30-nm-thick Ag film, strong reflection causes a standing wave. The amplitude difference between valleys and peaks is strongly related to the reflection. For the Ag/Si (30/15 nm) double-layered film, the standing wave with large amplitude differences between valleys and peaks can still be seen at 780 nm non-resonant wavelength. However, at 580 nm peak transmission wavelength, the reflection is significantly reduced and the amplitude differences between valleys and peaks in the standing wave are suppressed. The absorption loss is related to the imaginary part of medium permittivity and electric field amplitude by 
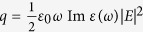
, where *ε*_0_ is vacuum permittivity and *ω* is operating frequency. The absorption profile across all layers is provided in Fig. 4c, indicating that most of the absorption occurs in the Si layer as opposed to the underlying Ag layer. At 520 nm wavelength where maximum absorption (~75%) and minimum reflection (~1%) occur, over 72% of the incident light is absorbed in the Si layer whereas only 3% of the light is absorbed in the Ag layer, with the remaining 24% of the light transmitted. At 580 nm wavelength where maximum transmission (~30%) occurs, over 53% of the incident light is absorbed in the Si layer whereas only 5% of the light is absorbed in the Ag layer, with the remaining 12% of the light reflected. Therefore, the transmission can be further enhanced by reducing the loss in the a-Si layer.

### Si/Ag/Si triple-layered film

To understand the dependence of transmission on the geometry of the proposed structure, we treat the Ag/Si double-layered film as a resonator and analyze its transmission via temporal coupled mode theory^28^,^29^. Energy exchanges among incident light, reflected light, transmitted light and the resonator is strongly correlated with the radiative decay process. The radiative decay rates relevant to intrinsic loss, reflection and transmission are represented as 1/*τ*_*i*_, 1/*τ*_*r*_ and 1/*τ*_*t*_, respectively. When the light with normalized power |*s*_*i*_|^2^ is incident into the system, the time derivative of the excited mode’s amplitude *a* can be expressed as:


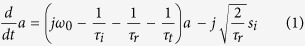


where *ω*_0_ is the resonant angular frequency. Given the equation connecting the incident field *s*_*i*_ and transmitted field *s*_*t*_, the transmission of the resonator can be denoted as:


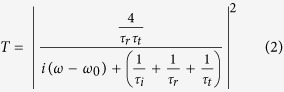


**Figure 5:**
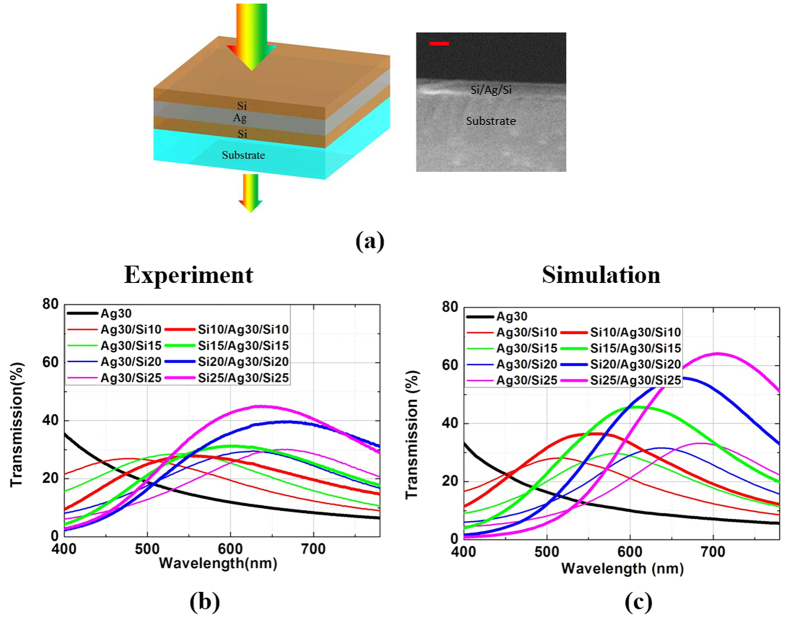
Symmetric Si/Ag/Si triple-layered film for further enhanced transmission. (**a**) A representation (both schematic diagram and SEM image of cross-section) of the proposed symmetric Si/Ag/Si triple-layered film. The scale bar is 100 nm. (**b**,**c**) are measured and simulated transmission of the proposed symmetric Si/Ag/Si triple-layered film at normal incidence, respectively.

Equation (2) indicates that the transmission *T* reaches the maximum at *τ*_*r*_ = *τ*_*t*_. Therefore, a symmetric structure can further enhance the transmission. Here another Si layer beneath the Ag layer is added to form a symmetric Si/Ag/Si triple-layered film, as illustrated in Fig. 5a. Figure 5a also presents the SEM image of the cross-section of the Si/Ag/Si triple-layered film. The boundary between the substrate layer and Si/Ag/Si layer is also clear. Fig. 5b,c show the experimental and simulated transmission for both Ag/Si non-symmetric double-layered and Si/Ag/Si symmetric triple-layered films with different Si thicknesses (30 nm Ag thickness), respectively. The experimental transmission, transmission enhancement factor, reflection and absorption for Si/Ag/Si triple-layered films with all four Ag thicknesses (20, 30, 40 and 50 nm) are presented in Figs S2 to S5 in the Supplementary Information, respectively. It can be distinctly seen that Si/Ag/Si symmetric configurations show higher optical transmission and broader windows than the Ag/Si asymmetric configuration. For example, the experimental maximum transmission for Si/Ag/Si (25/30/25 nm) symmetric film is 45% at 630 nm, well above that for Ag/Si (30/25 nm) asymmetric film (~30% at 660 nm). For the 50-nm-thick Ag film at 700 nm wavelength, the transmission is increased from 1.3% to 12% after coating two 25-nm-thick Si layers, exhibiting an over ninth enhancement in the transmission (Figs S2d and S3d). The simulated transmission is generally higher than that of corresponding measured transmission. This can be attributed to the light scattering loss induced by the rough film surfaces. Especially for triple-layered films, an extra rough film surface can lead to extra scattering loss.

### Transmission at oblique incidences

**Figure 6:**
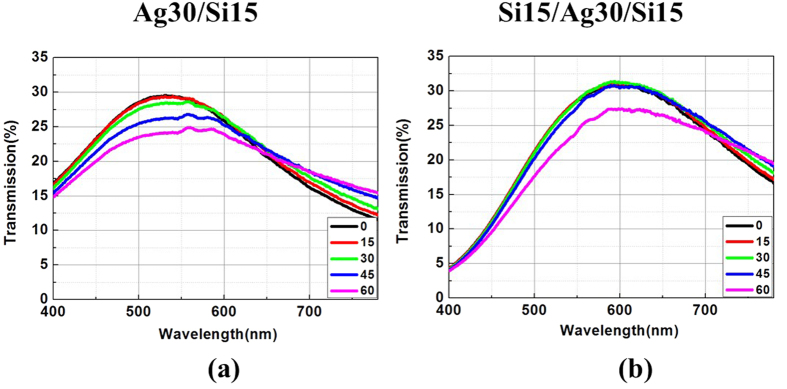
The measured angle resolved enhanced transmission spectra for both (**a**) Ag/Si (30/15 nm) double-layered and (**b**) Si/Ag/Si (15/30/15 nm) triple-layered films. The incident angles are 0°, 15°, 30°, 45° and 60° under the non-polarized light illumination.

To show the robustness of enhanced optical transmission of the proposed structures to oblique incidence, the measured angular resolved transmission spectra of two typical films (asymmetric Ag/Si (30/15 nm) and symmetric Si/Ag/Si (15/30/15 nm) films) for non-polarized light are provided in Fig. 6. For both films, the peak transmission is almost unaltered when the incident angle is increased to 30°. When the incident angle is increased to 45°, the peak transmission is only decreased by 5%. The robustness of enhanced optical transmission to the oblique incidence results from significantly suppressed propagation phase shift in the ultrathin Si layer. This is in sheer contrast to conventional *λ*/4 thickness film where the propagation phase shift dominates and thereby the enhanced transmission is sensitive to the incident angle.

### Performance as transparent metal electrodes

To show the conductivity of the proposed metallic film with enhanced transmission, the sheet resistances of Ag/Si films are characterized with a four-point probe and the results are provided in Table S1 in the Supplementary Information. Specifically, for the Si/Ag/Si and Ag/Si films with 30-nm-thick Ag, the sheet resistances are around 2 Ω/sq and 1.4 Ω/sq, respectively. All these values are much lower than those of commercial ITO films (around 60 Ω/sq for an 80 nm-thick ITO film^30^).

To quantify the optical performance of the Ag/Si and Si/Ag/Si as transparent metal electrodes for thin-film photovoltaics, we define a figure of merit (FOM) that denotes the ratio between the transmitted photon number and the total incident photons number from the sun. Here we assume crystalline silicon solar cell and effective wavelength for photovoltaics is from 400 to 1100 nm. The FOM can be denoted as:


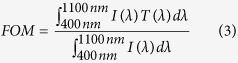


where *λ* is the wavelength, *I*(*λ*) is the AM 1.5G solar spectrum, and *T*(*λ*) is the transmission spectrum. Calculated FOMs for Ag/Si double-layered and Si/Ag/Si triple-layered films with various thicknesses provided in Table 1 enable an intuitive view on the transmission enhancement using metallic films with strong interference dominated by interface phase shift in solar cells. The FOM of the bare Ag film is 24.5%, 11.0% and 5.1% for 20-nm, 30-nm and 40-nm thickness, respectively. The FOM is improved by coating an a-Si layer, showing around 30% enhancement relative to the bare Ag film. For the Ag/Si double-layered film, there is an increase in the transmission with increasing Si thickness and then decrease at 25 nm Si thickness. As the Si thickness increases, the transmission peak wavelength red-shits, leading to an increasing FOM (Fig. 2); however, the transmission also decreases at short wavelength as the Si thickness increases further. Therefore, the FOM increases first and then decreases with Si thickness. The FOM for the Si/Ag/Si triple-layered film exhibits much higher enhancement relative to the bare Ag film. Specifically, the FOMs are 44.9%, 27.7%, and 14.4% for the triple-layered films (Si thickness 25 nm) with Ag thickness of 20 nm, 30 nm, and 40 nm, respectively, demonstrating 83.2%, 151.8% and 182.4% enhancement relative to those of the bare Ag film. The Si/Ag/Si triple-layered film enhances the transmission efficiency as well as the transmission bandwidth, thereby significantly enhance the FOM. For commercial ITO, the transmission is between 85%–90% between 400 and 1100 nm and the calculated FOM is around 85%, higher than those for the Ag/Si and Si/Ag/Si films. However, the ITO glass now suffers the high cost owing to limited availability of indium and new substitutes are highly desirable.

## Conclusions

In conclusion, we have demonstrated optical transmission enhancement utilizing strong interference in both Ag/Si double-layered and Si/Ag/Si triple-layered films. The strong resonance predominantly results from non-trivial phase shift at the metal-semiconductor interface, in sheer contrast with conventional interference where transmission phase shifts dominate, thereby leading to significantly reduced film thickness. This strong interference induced transmission enhancement also exhibits broadband and wide-angle operation. The Ag/Si double-layered and Si/Ag/Si triple- layered films also show much lower resistances than their counterparts for bare Ag films. Considering one of its potential applications as a transparent metal electrode in a solar cell, a calculated 182.4% enhancement in the total transmission efficiency relative to that of a single metallic film is expected. The simplicity and flexibility of planar layered structures and their fabrication methods promise new routes toward the development of large-area metallic films with high transmission for a variety of energy-harvesting applications such as thin-film photovoltaics and surface photocatalysis.

## Methods

### Experiment

The Ag and a-Si are deposited in the chamber of the high-vacuum sputtering system. During the Ag deposition, the Argon pressure in the chamber is set to 0.15 Pa with no heating on the substrate. The deposition rates for Ag and a-Si are 6.7 A/s and 3A/s, respectively. The a-Si deposition rate is controlled by the radio frequency power and the Ag deposition rate is controlled by the DC power.

### Simulation

Throughout the paper, the theoretical reflection (*R*) and transmission (*T*) spectra are calculated using characteristic matrix method for each film, as shown in ref. 1. The absorption is obtained by *A* = 1−*R*−*T.* The field pattern (Fig. 4) of the nanostructure is modeled using the commercial software COMSOL MULTIPHYSICS based on finite element method.

## Additional Information

**How to cite this article**: Li, Q. *et al*. Transmission enhancement based on strong interference in metal-semiconductor layered film for energy harvesting. *Sci. Rep.* **6**, 29195; doi: 10.1038/srep29195 (2016).

## Supplementary Material

Supplementary Information

## Figures and Tables

**Table 1:** FOMs of Ag, Ag/Si and Si/Ag/Si films.

Ag Thickness (nm)	FOM (%)	Ag/Si Thickness (nm)	FOM (%)	Si/Ag/Si Thickness (nm)	FOM (%)
20	24.5	20/10	31.4	10/30/10	36.4
		20/15	32.1	15/30/15	40.1
		20/20	32.2	20/30/20	43.1
		20/25	31.7	25/30/25	44.9
30	11.0	30/10	15.5	10/30/10	19.9
		30/15	15.9	15/30/15	22.9
		30/20	15.9	20/30/20	25.6
		30/25	15.6	25/30/25	27.7
40	5.1	40/10	7.4	10/40/10	10.0
		40/15	7.5	15/40/15	11.6
		40/20	7.4	20/40/20	13.2
		40/25	7.2	25/40/25	14.4
