# Supplementary material for: Transmission enhancement based on strong interference in metal-semiconductor layered film for energy harvesting
Source: Sci Rep. 2016 Jul 12;6:29195. doi: 10.1038/srep29195 (PMC4940859; doi:10.1038/srep29195)
Supplement: Supplementary Information [file srep29195-s1.pdf]

Supplementary Information

Transmission enhancement based on strong interference in metal-semiconductor layered film for energy harvesting

Qiang Li, Kaikai Du, Kening Mao, Xu Fang, Ding Zhao, Hui Ye and Min Qiu

State Key Laboratory of Modern Optical Instrumentation, College of Optical Science and Engineering, Zhejiang University, Hangzhou 310027, China,

1 The transmission photographs of the fabricated Ag/Si double-layered films

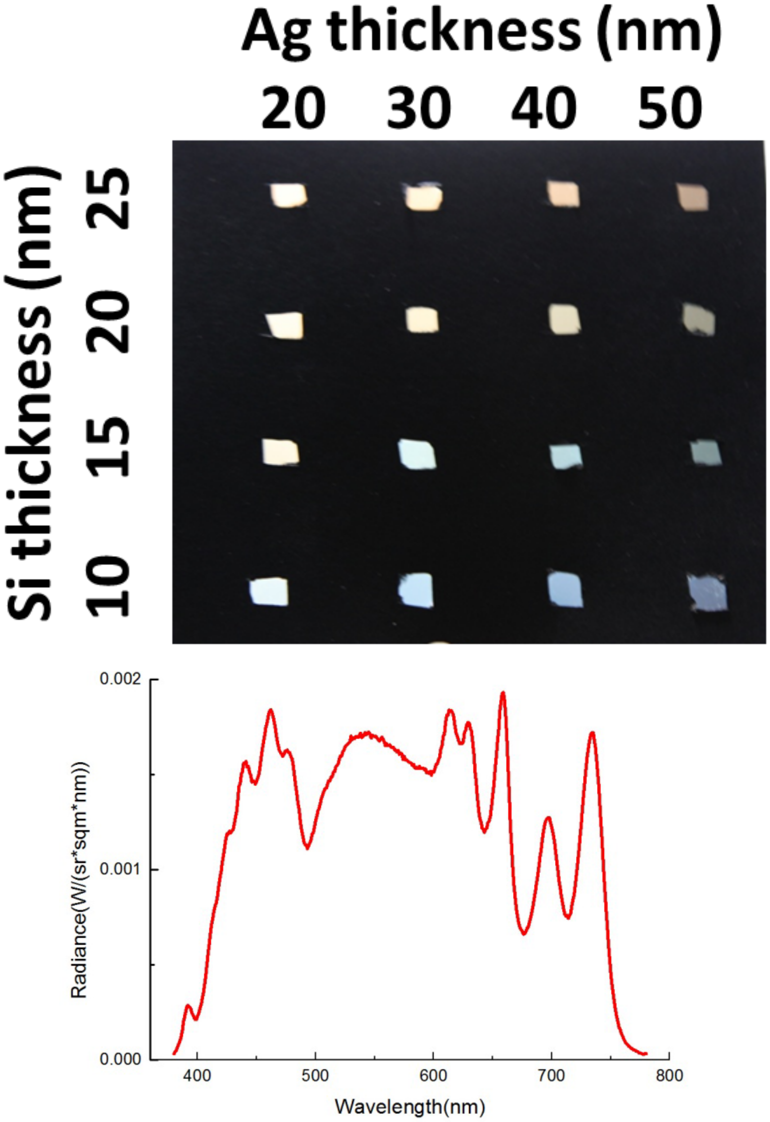

Figure S1. (a) The transmission photographs of the fabricated Ag/Si double-layered films (16 samples in total). From left to right, the Ag thickness is 20 nm/30 nm/40 nm/50 nm. From top to bottom, the Si thickness is 25 nm/20 nm/15 nm/10 nm. (2) The spectrum of light used for transmission imaging.

2 The measured transmission at normal incidence for Ag/Si double-layered and Si/Ag/Si triple-layered films

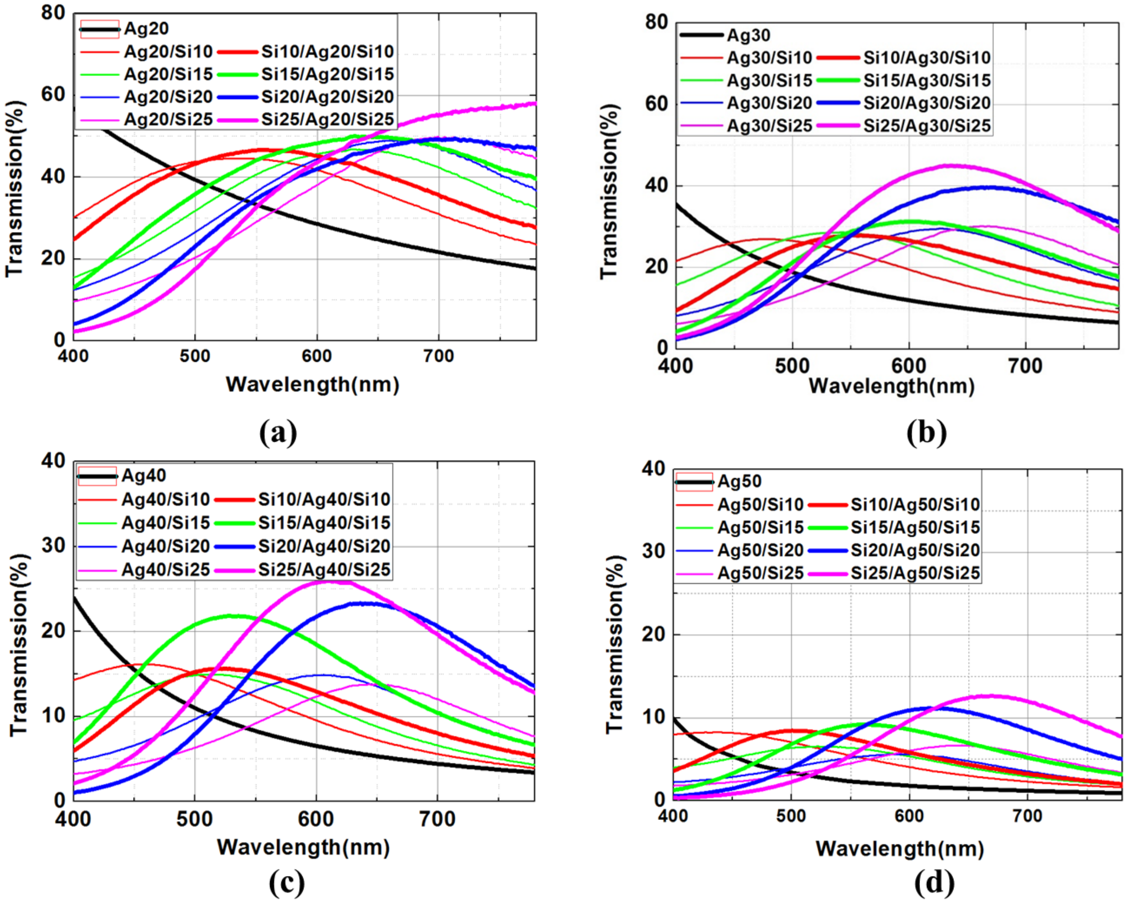

Figure S2. The measured transmission at normal incidence for Ag/Si double-layered and Si/Ag/Si triple-layered films. The Ag thicknesses are 20 nm, 30 nm, 40 nm and 50 nm, respectively.

3 The measured transmission enhancement factor at normal incidence for Ag/Si double-layered and Si/Ag/Si triple-layered films

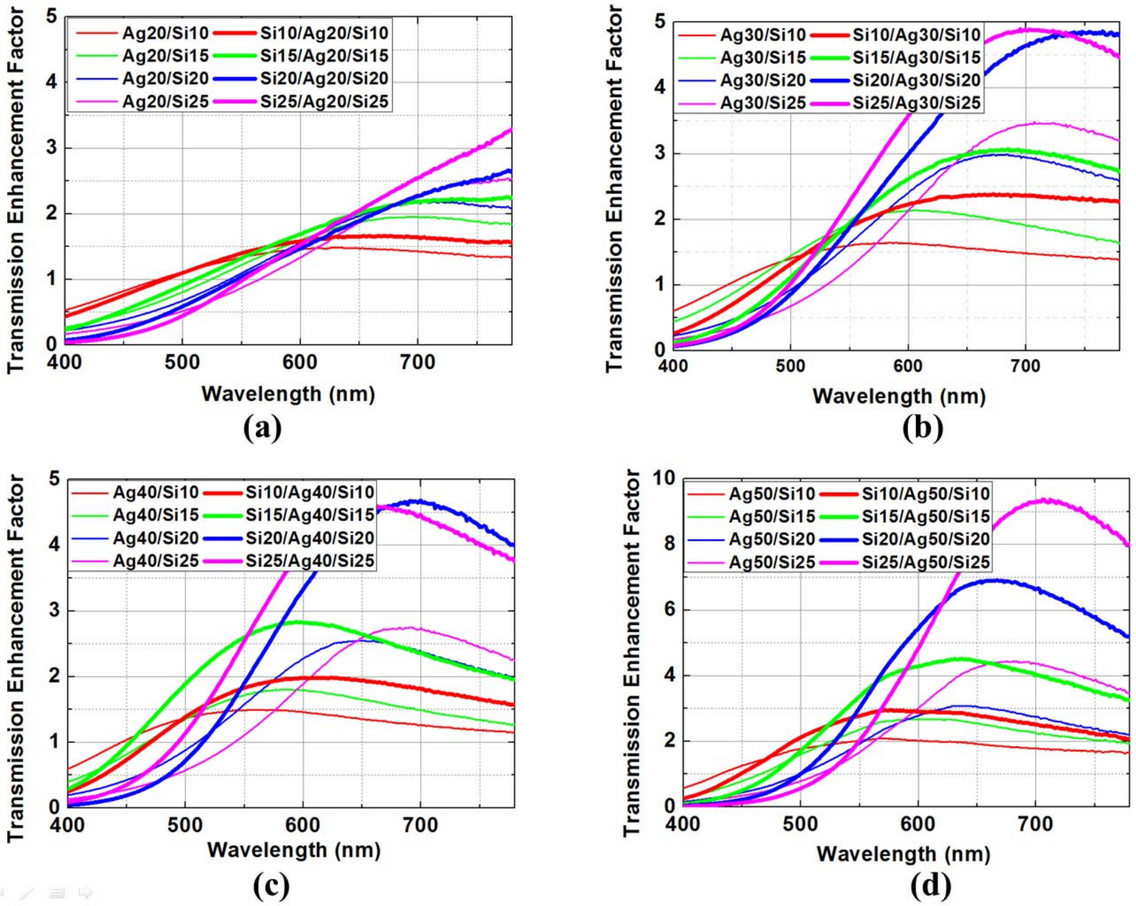

Figure S3. The measured transmission enhancement factor at normal incidence for Ag/Si double-layered and Si/Ag/Si triple-layered films. The Ag thicknesses are 20 nm, 30 nm, 40 nm and 50 nm, respectively.

4 The measured absorption at normal incidence for Ag/Si double-layered and Si/Ag/Si triple-layered films

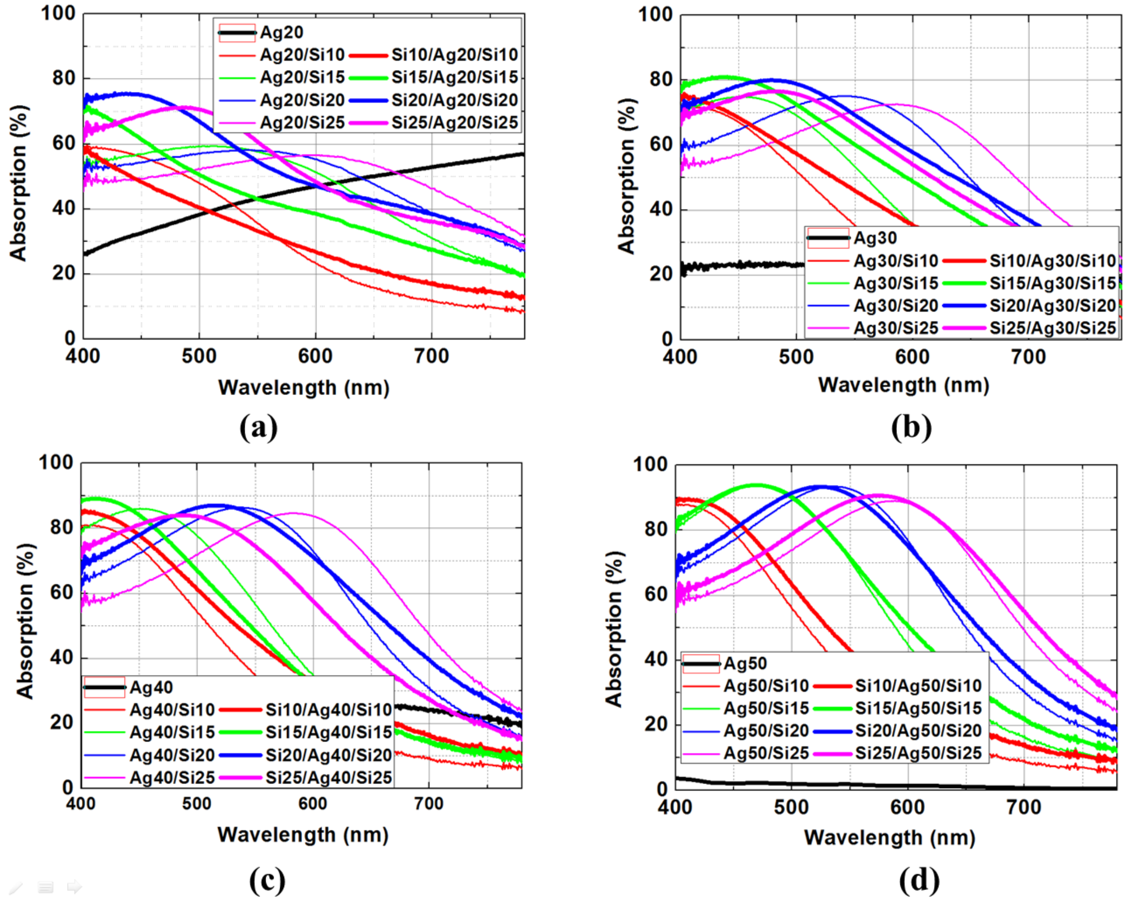

Figure S4. The measured absorption at normal incidence for Ag/Si double-layered and Si/Ag/Si triple-layered films. The Ag thicknesses are 20 nm, 30 nm, 40 nm and 50 nm, respectively.

5 The measured reflection at normal incidence for Ag/Si double-layered and Si/Ag/Si triple-layered films

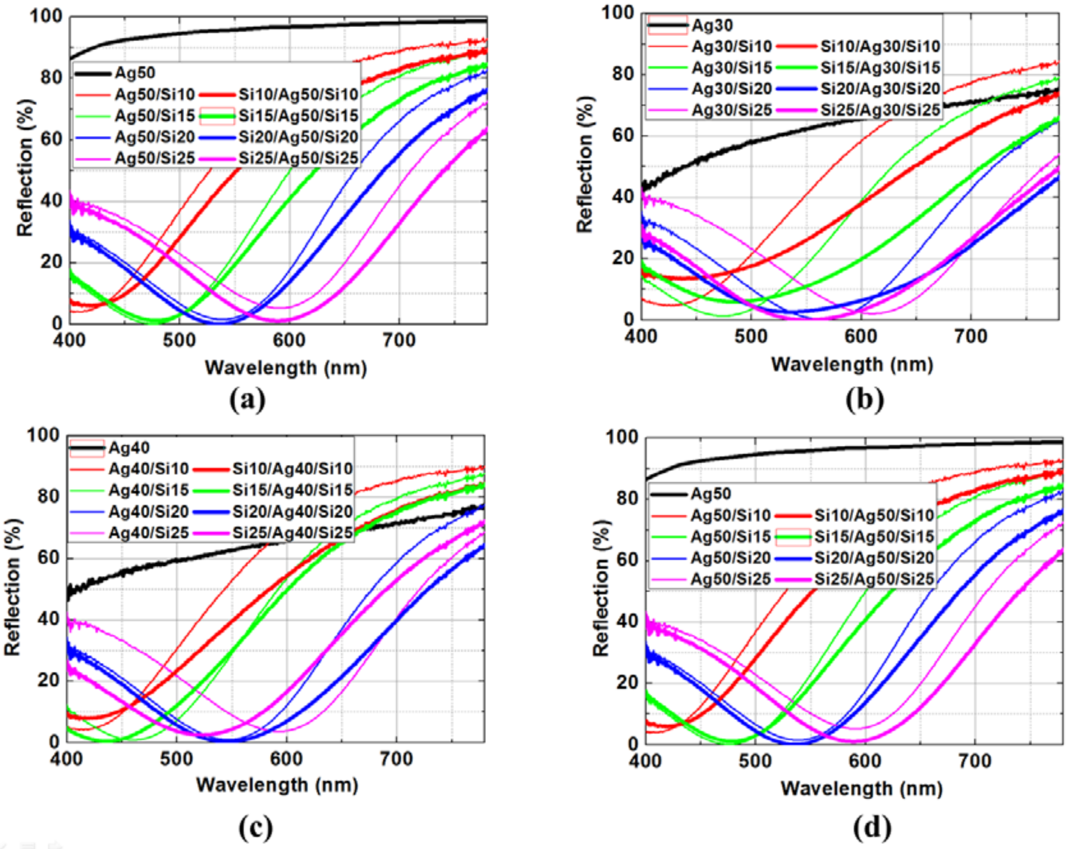

Figure S5. The measured reflection at normal incidence for Ag/Si double-layered and Si/Ag/Si triple-layered films. The Ag thicknesses are 20 nm, 30 nm, 40 nm and 50 nm, respectively.

6 The measured sheet resistance of Ag/Si and Si/Ag/Si films

For Ag/Si and Si/Ag/Si films, the sheet resistances are characterized on the bottom Ag layers and bottom Si layers, respectively. The measured sheet resistances for Si/Ag/Si films are larger than those of Ag/Si films owing to different layers used in the characterization.

Table S1 Resistances of Ag/Si and Si/Ag/Si films

Ag/Si Thickness (nm)	Resistance (Ω/sq)	Si/Ag/Si Thickness (nm)	Resistance (Ω/sq)
30/10	1.38	10/30/10	2.01
30/15	1.45	15/30/15	1.81
30/20	1.42	20/30/20	2.11
30/25	1.49	25/30/25	2.00
40/10	0.99	10/40/10	1.26
40/15	0.97	15/40/15	1.20
40/20	0.95	20/40/20	1.35
40/25	0.89	25/40/25	1.10
